# Comparative study of protein-protein interaction observed in PolyGalacturonase-Inhibiting Proteins from *Phaseolus vulgaris *and *Glycine max *and PolyGalacturonase from *Fusarium moniliforme*

**DOI:** 10.1186/1471-2164-10-S3-S19

**Published:** 2009-12-03

**Authors:** Aditi Maulik, Hiren Ghosh, Soumalee Basu

**Affiliations:** 1 Department of Bioinformatics, School of Biotechnology, West Bengal University of Technology BF-142, Salt Lake, Kolkata 700064, India

## Abstract

**Background:**

The PolyGalacturonase-Inhibiting Proteins (PGIP) of plant cell wall limit the invasion of phytopathogenic organisms by interacting with the enzyme PolyGalacturonase (PG) they secrete to degrade pectin present in the cell walls. PGIPs from different or same plant differ in their inhibitory activity towards the same PG. PGIP2 from *Phaseolus vulgaris *(*Pv*) inhibits the PG from *Fusarium moniliforme *(*Fm*) although PGIP1, another member of the multigene family from the same plant sharing 99% sequence similarity, cannot. Interestingly, PGIP3 from *Glycine max *(*Gm*) which is a homologue of PGIP2 is capable of inhibiting the same PG although the extent of similarity is lower and is 88%. It therefore appears that subtle changes in the sequence of plant PGIPs give rise to different specificity for inhibiting pathogenic PGs and there exists no direct dependence of function on the extent of sequence similarity.

**Results:**

Structural information for any PGIP-PG complex being absent, we resorted to molecular modelling to gain insight into the mechanism of recognition and discrimination of PGs by PGIPs. We have built homology models of *Pv*PGIP1 and *Gm*PGIP3 using the crystal structure of *Pv*PGIP2 (1OGQ) as template. These PGIPs were then docked individually to *Fm*PG to elucidate the characteristics of their interactions. The mode of binding for *Pv*PGIP1 to *Fm*PG considerably differs from the mode observed for *Pv*PGIP2-*Fm*PG complex, regardless of the high sequence similarity the two PGIPs share. Both *Pv*PGIP2 and *Gm*PGIP3 despite being relatively less similar, interact with residues of *Fm*PG that are known from mutational studies to constitute the active site of the enzyme. *Pv*PGIP1 tends to interact with residues not located at the active site of *Fm*PG. Looking into the electrostatic potential surface for individual PGIPs, it was evident that a portion of the interacting surface for *Pv*PGIP1 differs from the corresponding region of *Pv*PGIP2 or *Gm*PGIP3.

**Conclusion:**

van der Waals and eletrostatic interactions play an active role in PGIPs for proper recognition and discrimination of PGs. Docking studies reveal that *Pv*PGIP2 and *Gm*PGIP3 interact with the residues constituting the active site of *Fm*PG with implications that the proteins bind/block *Fm*PG at its active site and thereby inhibit the enzyme.

## Background

Plants are under constant threat of infections caused by pathogens that range from viruses, bacteria, fungi to nematodes and insects. The efficacy of plant defense depends on its ability to recognize a pathogen and mount the appropriate defense response. Plants often employ cell surface and intracellular receptors to detect a pathogen associated molecular pattern (PAMP) and trigger immune response against the invader. PolyGalacturonase-Inhibiting Protein (PGIP) is one among the pathogenesis-related (PR) proteins that is found at the cell surface of plant cells. It binds and inhibits the enzyme PolyGalacturonase (PG) from the invading pathogen which could be fungus, insect or bacterium, thus preventing its colonization in the host cell and hence the progress of the disease [1,2]. PolyGalacturonases (PG) are a class of pectinolytic enzyme secreted by the pathogen at the early stages of infection to depolymerize homogalacturonan (HGA), the main component of pectin in the plant cell wall [3]. HGA is the 1, 4 linked alpha-D-galactosyluronic acid polymer found in the plant cell which forms the first line of barrier and thereby plays a critical role in controlling pathogen invasion [4-7].

The interaction of the plant protein PGIP with the fungal or insect PG limits the destructive potential of the PG and leads to the accumulation of elicitor active oligogalacturonides as shown by in vitro studies [8]. Hence PGIP seems to exert a dual role during fungal attack. It limits pathogen penetration and tissue colonization by inhibiting PG activity, this in turn, favours the accumulation of oligogalacturonides, which activate a prompt defense response [9].

**Figure 1:**
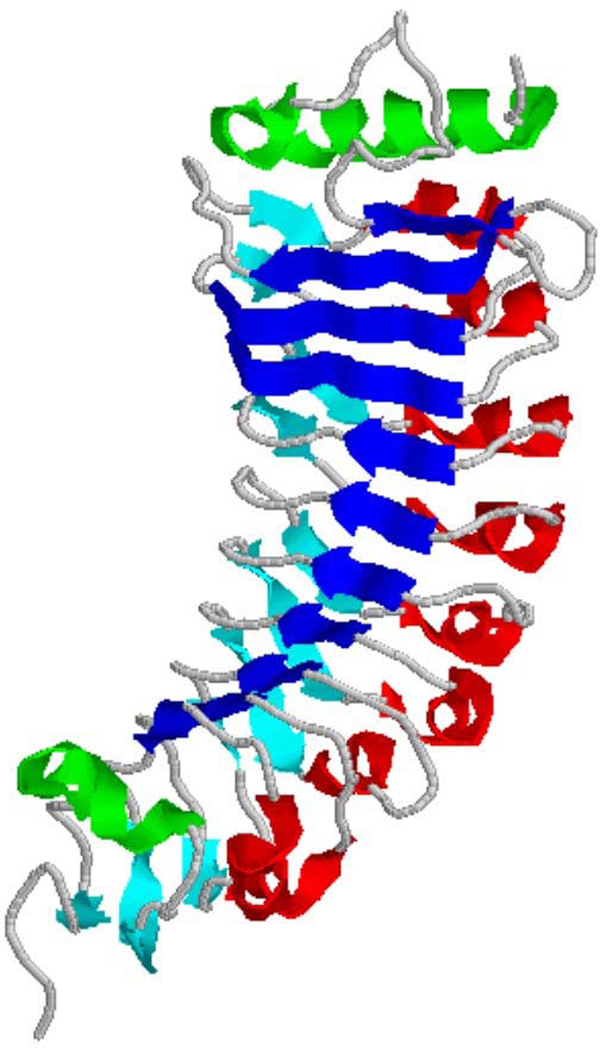
**Ribbon representation of Crystal structure of *Pv*PGIP2 (**1OGQ**)**. The protein is composed of tandemly repeating units called LRRs. The N-terminal and C-terminal cysteine-rich domains are shown in green. Three structural elements characterize this fold. The β-sheet B1 occupies the concave face of the scaffold (blue), a regular array of 3_10 _helices characterizes the convex face (red), and a second non-regular β-sheet (B2; cyan) is found between the two faces.

PGIPs belong to the Leucine-rich Repeat (LRR) super family of proteins [10] containing tandem repeats of a 20-30 amino acid stretch of the extracytoplasmic type with a consensus that bear a conserved part [LxxLxLxxNxL or LxxLxLxxCxxL (L = I, L, V, F; N = N, T, S, C; C = C, S; x = any amino acid)] and a variable part [11,12]. The LRR-fold (Figure 1) which is believed to be specialized in protein-protein interaction is quite extensively used for the immune functions and for the recognition of non-self molecules by plants. The crystal structure of the only PGIP (PGIP2 from *Phaseolus vulgaris*) as well as the first LRR protein belonging to the plant-specific subfamily [13] ([ILVF]xx[ILVF]xx[ILVF]x[ILVF]xx[NTSC]x[ILVF] [TS]GxIPxx[ILVF]Gx) reveals a typical curved and elongated shape. Eight β strands (with one long β strand, B1, at the N-terminal end) comprise the inner concave face of the curved surface. On the opposite side of the β sheet, there are nine 3_10 _helices that are almost parallel to the β sheet. The concave surface is known to bear residues necessary for binding and recognition specificity in this class of protein [14].

PGIPs function as very specific inhibitors [15]. PGIP from a particular plant that is able to inhibit the PG from a certain pathogen might not be able to inhibit PGs from other pathogens. Besides, for cases when a PGIP is able to inhibit PGs from different pathogens, the percent inhibition differs. Thus in plants, small gene families encode PGIP isoforms that differ in affinity and specificity for PGs secreted by different pathogens [1]. Recognition specificity results from a few variations in the amino acid sequence of the PGIPs [16]. Plants successfully defend themselves from the attack of a wide range of PGs from pathogenic microorganisms through the role played by protein-protein interaction involving these PGIP proteins.

Significant contributions have been made with experimental studies on the different isoforms of PGIP from a variety of plants. It has been seen that the overexpression of two PGIP genes, *Atpgip1 *and *Atpgip2 *genes from *Arabidopsis thaliana *limits the colonization by *B. cinerea *and also reduces the disease symptoms [17]. In transgenic tomato and grapevine plants, a significant increase of PG-inhibitory activity and a decrease in susceptibility to *B. cinerea *have been observed. Plant resistance have been successfully enhanced by the overexpression of pear PGIP in tomato [18] and *Vitis vinifera *[19], and also bean PGIP in tobacco [20]. Accumulation of information on several of the properties exhibited by PGIPs have generated an interest in exploiting them as tools for enhancing plant resistance. In this regard, PGIPs of bean plant (*Phaseolus vulgaris*) needs special mention.

Bean PGIPs are encoded by a family of genes [21], the products of which are *Pv*PGIP1, *Pv*PGIP2, *Pv*PGIP3 *and Pv*PGIP4. All of these show distinct regulation and specificity exhibiting different inhibitory capabilities against the PGs of *Botrytis cinerea*, *Colletotrichum gloeosporioides, Stenocarpella maydis, Fusarium moniliforme and Aspergillus niger *[21]. The four mature products of the pgip genes have a very high extent of sequence similarity differing between 8 to 81 amino acids. Phylogenetic analysis indicates that *Pvpgip1-Pvpgip2 *groups with *Gmpgip3 *of soybean (*Glycine max*), a species of the Phaseoleae tribe close to bean, which suggests that these two genes are probably closer to the ancestral gene than are *Pvpgip3 *and *Pvpgip4 *[7]. Interestingly, although PGIP1 and PGIP2 from *Phaseolus vulgaris *share 99% sequence similarity, *Pv*PGIP2 has been reported to inhibit PG from *Fusarium moniliforme *(*Fm*) while *Pv*PGIP1 does not [16]. Strikingly, *Gm*PGIP3 of soybean (*Glycine max*), shares a much lesser similarity of 88% with *Pv*PGIP2 and yet has the ability to inhibit *FmPG *with an inhibition kinetics similar to that of *Pv*PGIP2 [22]. It is thus evident that the ability of PGIP molecules to defend plants against infection is not buried in the extent of similarity shared between the sequences alone. Probable implications could be the presence of key structural features important for the necessary protein-protein interaction that need to be conserved to preserve the function.

It would be interesting to study the molecular interaction of different PGIPs sharing different range of sequence variation, with the same PG molecule to elucidate the relation between the extent of sequence similarity and the corresponding ability/inability to inhibit PG. Three PGIPs, *Pv*PGIP1 and *Pv*PGIP2 from *Phaseolus vulgaris *and *Gm*PGIP3 from *Glycine max *have been chosen for this purpose keeping in mind that they share a high level of sequence similarity and yet show distinct difference in their function as expressed through their ability/inability to inhibit the same PG from *Fusarium moniliforme *(*Fm*PG). In this study, homology models of two PGIP molecules namely *Pv*PGIP1 and *Gm*PGIP3 have been built using the crystal structure of *Pv*PGIP2 as the template. The crystal structure of *Fm*PG being available, the three PGIPs have been subsequently docked to *Fm*PG. In order to elucidate the mechanism underlying and the strategy undertaken by the plants in utilizing the different isoforms of PGIP, a comparative study of the interaction between these three pairs namely *Pv*PGIP1 and *Fm*PG, *Pv*PGIP2 and *Fm*PG and *Gm*PGIP3 and *Fm*PG have been accomplished. A comparison of the binding modes, the interacting residues and the electrostatic potential surface along the interface of the PGIP-PG complex have been investigated. Change in intermolecular distances, hydrogen bond formation and electrostatic surface potential upon *in silico *mutation of *Pv*PGIP2 have also been explored.

## Results and discussion

### Sequence analysis

#### Domain search

**Figure 2:**
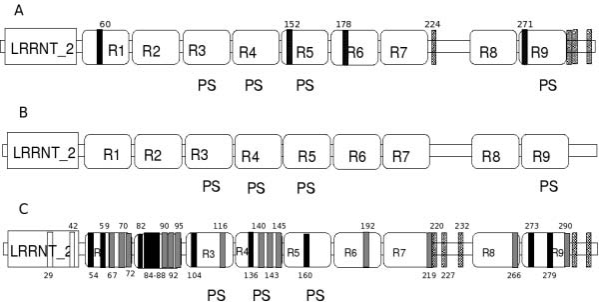
**Domain architecture (DA) of the three PGIP molecules**. A, B & C show the DA of *Pv*PGIP1, *Pv*PGIP2 and *Gm*PGIP3 respectively. Domains in all the three proteins are arranged as one LRR_NT2 domain followed by nine leucine-rich repeats (R1-R9). The domains are shown by rectangular boxes. Out of the nine LRRs, four have been marked as plant-specific (PS) LRRs (R3-R5, R9). The vertical bars in A and C denote the sequence variations with *Pv*PGIP2 and the numbers stand for the residue number. The types of vertical bars change with the position of the varying residue, an information obtained from the multiple sequence alignments shown in Figure 3. Plain vertical bars stand for changes that are found in the LRR_NT2 region, the black and grey bars signify residue positions belonging to the conserved region and the variable region of LRRs respectively, while the hatched bars are for those in the non-LRR region.

Domain search using Pfam [23] discloses that the three PGIP molecules (two from *P. vulgaris *and one from *G. max*) studied here share identical domain architecture consisting of one LRR_NT2 (a domain found specifically at the N terminal ends of leucine-rich repeat proteins) at the N-terminal end and nine leucine-rich repeats (R1-R9) (Figure 2). Out of the nine LRRs, four have been identified as plant-specific (PS) LRRs (R3-R5, R9) using EMBOSS [24] with the pattern [ILVF]xx[ILVF]xx[ILVF]x[ILVF]xx[NTSC]x[ILVF] [TS]GxIPxx[ILVF]Gx.

#### Pairwise alignment

Pairwise alignment carried out in order to identify similarities between the PGIP molecules at the sequence level, shows only eight varying residue positions between *Pv*PGIP1 and *Pv*PGIP2 (shown by the vertical bars in Figure 2A) and 34 varying positions between *Pv*PGIP2 and *Gm*PGIP3 (shown in Figure 2C). Four out of the eight variations between *Pv*PGIP1 and *Pv*PGIP2 fall in the repeat regions and the rest reside in the non-LRR (intervening region between two repeats) region of the proteins. Two (R5 and R9) out of the four PS-LRRs and the first (R1) and the sixth (R6) repeat harbour these four variations mentioned above. The 34 variations between *Pv*PGIP2 and *Gm*PGIP3 on the other hand, are divided amongst all domains including LRRNT_2 and all the plant specific LRRs (Figure 2C).

#### Distribution of the variations in the domains

**Figure 3:**
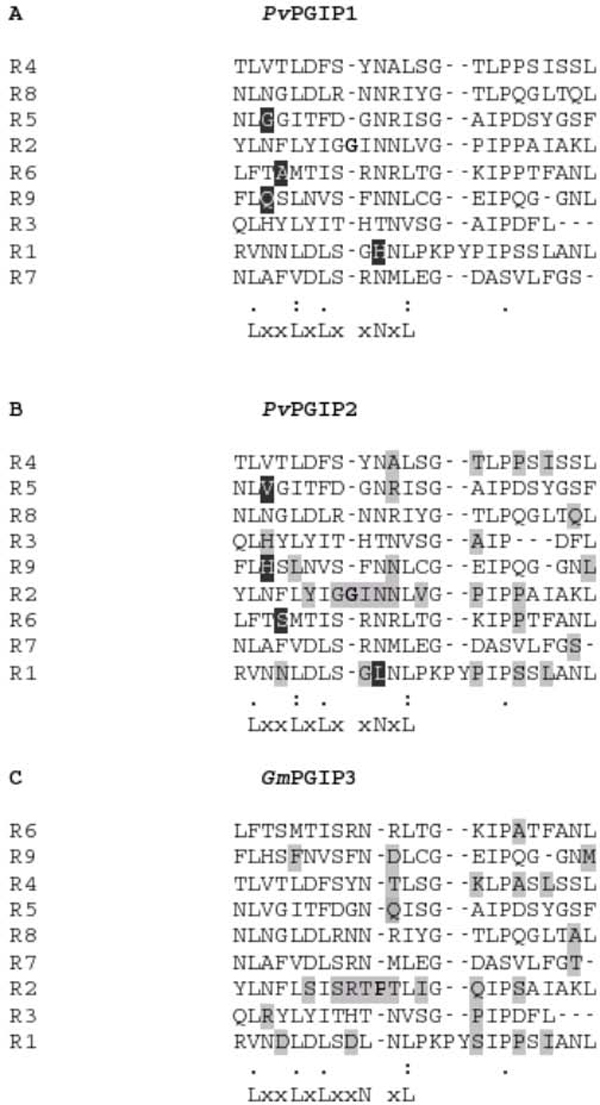
**Three individual multiple sequence alignments of the nine repeats of the PGIP molecules**. A, B and C show the multiple sequence alignments with the nine LRR repeats (R1-R9) for *Pv*PGIP1, *Pv*PGIP2 and *Gm*PGIP3 respectively. The black coloured positions in A and B correspond to the residues that differ between *Pv*PGIP1 and *Pv*PGIP2 while grey coloured positions in B and C highlight the varying residues between *Pv*PGIP2 and *Gm*PGIP3. The varying residue positions that are in bold are considered as insertion.

Out of the 34 residues that differ in *Gm*PGIP3 from *Pv*PGIP2 only two changes (Position 297 and 311 in Figure 1) are common with the 8 differing residues between *Pv*PGIP1 and *Pv*PGIP2 leaving the rest 32 variations to be at unique positions. 27 out of these 34 fall in the LRR regions and 2 in the LRRNT_2 region leaving altogether 5 positions to remain scattered in the intervening non-LRR regions. The sequence variation that fall in the LRR region in *Pv*PGIP1(4) and *Gm*PGIP3(27) with *Pv*PGIP2 could be distributed amongst the conserved portion of the repeat or the variable portion of it. It becomes an important task to determine this, as there are positions in the repeats where sequence variation is accepted without an associated change in structure. To account for the distribution of these variations with respect to different regions in the repeats, we constructed individual multiple sequence alignments. Figure 3 shows the multiple sequence alignment of nine repeats each from *Pv*PGIP1, *Pv*PGIP2 and *Gm*PGIP3. The conserved region of the repeats are marked by the conserved pattern of LRRs (written below each alignment) while the rest of the repeat signify its variable region. Considering an insertion of a residue (G) in R2 for *Pv*PGIP1 and *Pv*PGIP2 and an insertion of P in R2 of *Gm*PGIP3 at another position, the variations (marked by black rectangles for those between *Pv*PGIP2 and *Pv*PGIP1 and grey rectangles for those between *Pv*PGIP2 and *Gm*PGIP3) can be rightly explained to be concentrated in positions (mostly X or variable region) where the motif itself has the provision for changes. The details of the distribution of the variations (8 for *Pv*PGIP1 and 34 for *Gm*PGIP3) in the LRR (conserved (**c**) and variable (**v**) region) and the non-LRR regions with reference to the residue type is tabulated in the Additional file 1. Depending on whether the variation is in the conserved or variable part of the repeat or in the non-LRR region, black, grey and hatched vertical bars are respectively used in Figure 2A and 2C. In both these proteins (*Pv*PGIP1 and *Gm*PGIP3 that share a similarity of 87%), most of the changes that fall in the LRR region (black bars in Figure 2) are either an allowed variant of the conserved position (L or C or N) or is any amino acid in the X position of the conserved region or is an amino acid belonging to the variable region of the repeat (grey bars). One exception is the variation in the conserved position N of R1 in *Pv*PGIP1 (Figure 3A) where the replacing amino acid H is not an allowed variant. Regarding the variations in the non-LRR region, Additional file 1 points out that whereas *Pv*PGIP1 and *Pv*PGIP2 share 50% of the changes in this region, *Gm*PGIP3 and *Pv*PGIP2 show only 15% of it. This could probably indicate specific regions of importance in PGIPs for the introduction of variation for recognition of different enzymes from pathogens.

#### Modelling

The crystal structure of *Pv*PGIP2 (1OGQ) being available and also since *Pv*PGIP1 and *Pv*PGIP2 as well as *Pv*PGIP2 and *Gm*PGIP3 share high percentage of sequence similarity, homology models of *Pv*PGIP1 and *Gm*PGIP3 were built using *Pv*PGIP2 as template. One model each for *Pv*PGIP1 and *Gm*PGIP3 were selected using PROCHECK [25]. In each case, the model that was chosen supported the following criteria:

(i) more number of residues in the core region

(ii) no residues in the disallowed region

These models of *Pv*PGIP1 and *Gm*PGIP3 were solvated in water and were subjected to energy minimization followed by molecular dynamics simulation of 1 ns. A similar scheme of equilibration was taken up for the crystal structure of *Pv*PGIP2. RMSD of the equilibrated structures of *Pv*PGIP1 and *Gm*PGIP3 were calculated with respect to the equilibrated structure of *Pv*PGIP2 and was found to be 1.45Å and 1.66Å respectively.

#### Docking

We used GRAMM-X [26] for docking three pairs of proteins in order to unveil the mode of interaction within and across each pair. This docking program executes a rigid-body search using Fast Fourier Transform (FFT) correlation with simplified geometry employing shape complementarity and hydrophobicity in the scoring function. In this study we have used GRAMM-X as a protein-protein docking program as it extends the GRAMM Fast Fourier Transformation methodology by applying smoothed Lennard-Jones potential, refinement stage and knowledge-based scoring, thus giving rise to best surface match. The output of GRAMM-X is a PDB file containing the structures of ten models ranked as the most probable prediction candidate, according to the scoring function used. The cell-wall degrading enzyme PG, from the fungus *Fusarium moniliforme *was common in all the pairs and the other three molecules include *Pv*PGIP1, *Pv*PGIP2 and *Gm*PGIP3. The crystal structure of *Fm*PG (1HG8) used in this study had also been solvated in water and was then subjected to energy minimization followed by molecular dynamics (MD) of 1 ns.

#### Docking studies of *Fm*PG-*Pv*PGIP2

**Figure 4:**
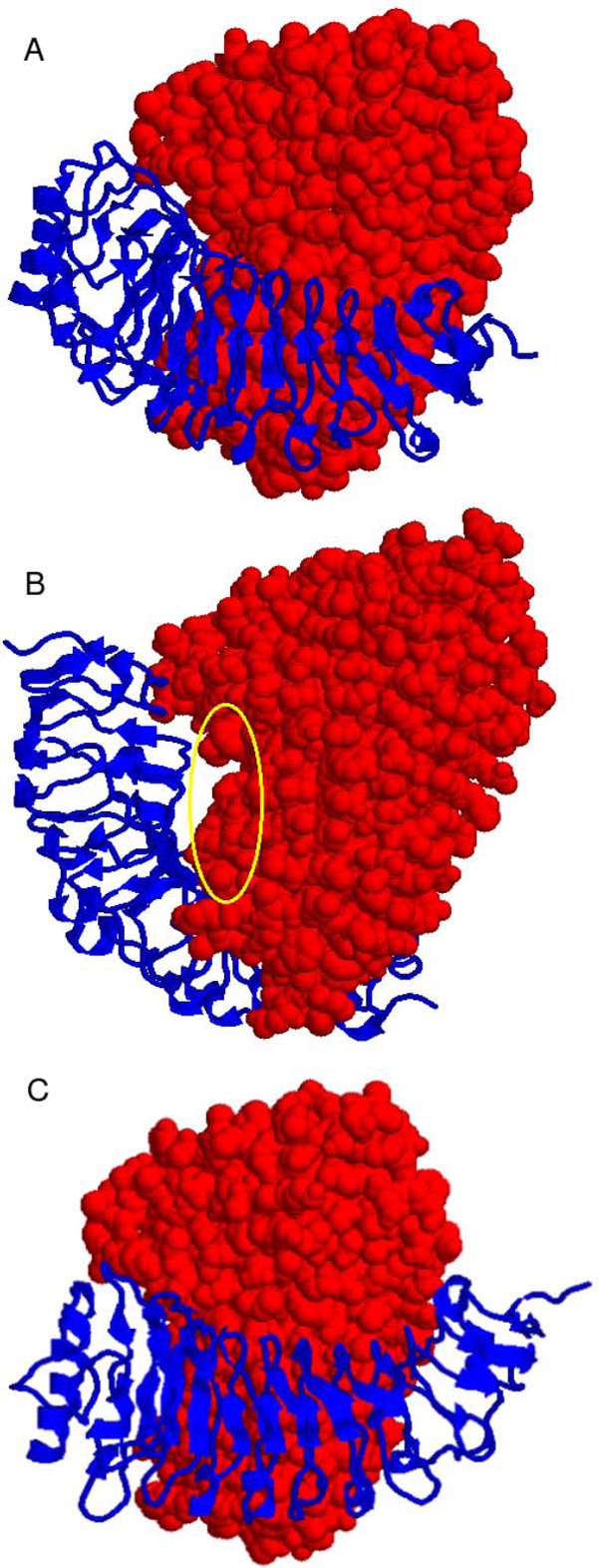
**Docked complexes of PGIP (ribbon representation in blue) and *Fm*PG (space-filling representation in red)**. A and C show how *Pv*PGIP2 (blue) and *Gm*PGIP3 (blue) respectively, hinder the substrate binding site and block the active site cleft of *Fm*PG (red). The only model of the *Pv*PGIP1-*Fm*PG complex where *Pv*PGIP1 (blue) docks near the active site of *Fm*PG (red) although not blocking it unlike the other two complexes, is shown in B. The marked area shown by the yellow ellipse in B indicates the portion of *Fm*PG that *Pv*PGIP1 has been unable to block.

It has been reported that since *Pv*PGIP2 inhibits *Fm*PG, hence one would expect *Pv*PGIP2 to interact with PG by blocking the active site and/or the substrate binding cleft of the fungal enzyme. We obtained top scoring ten docked complexes of *Pv*PGIP2-*Fm*PG from GRAMM-X. One model in the exact expected orientation was selected on the basis of the existing experimental studies (see below). In the docked complex (Figure 4A), *Pv*PGIP2 has covered the active site cleft by hindering the substrate binding site of *Fm*PG. This is evident from the change in Solvent Accessible Surface Area (SASA) (shown in Table 1) for the residues Asp-167, Asp-188, Asp-189, Arg-243, and Lys-245 of *Fm*PG that are known to be located inside the deep cleft and form the putative active site [27]. Functional studies confirm the involvement of these residues in the enzymatic reaction mechanism and or binding to the substrate which is in agreement with the reported mode of competitive inhibition with *Fm*PG [27].

**Table 1:** Change in Solvent Accessible Surface Area (SASA) in PGIP and PG upon complexation: The table summarizes the change in solvent accessible surface area of some biologically important residues of *Pv*PGIP2-*Fm*PG and *Gm*PGIP3-*Fm*PG before and after complexation.

Change in SASA (in nm^2^) upon complexation of *Pv*PGIP2 with *Fm*PG			
**Residues in *Pv*PGIP2**			

**Residue No.**	**Residue name**	**SASA in *Pv*PGIP2**	**SASA in complex**

152	V	0.726572	0.363286
178	S	0.264561	0.11834
224	Q	0.496097	0.137248
271	H	1.01276	*0.585858*

**Residues in *Fm*PG**			

**Residue No.**	**Residue name**	**SASA in *Pv*PGIP2**	**SASA in complex**

167	D	0.348913	0.306561
188	D	0.381939	0.141431
189	D	0.150885	0.0896238
243	R	0.158454	0.0127235
245	K	0.403263	*0.510823*

**Change in SASA (in nm^2^) upon complexation of *Gm*PGIP3 with *Fm*PG**			

**Residues in *Gm*PGIP3**			

**Residue No.**	**Residue name**	**SASA in *Gm*PGIP3**	**SASA in complex**

152	V	0.396312	0.066052
178	S	0.4249	0.182517
224	Q	0.517371	0.29529
225	K	0.6919	0.1325
271	H	1.21099	0.712258

**Residues in *Fm*PG**			

**Residue No.**	**Residue name**	**SASA in *Gm*PGIP3**	**SASA in complex**

167	D	0.348913	0.292443
188	D	0.381939	0.207483
189	D	0.150885	0.033026
243	R	0.158454	0.0254469
245	K	0.403263	0.523546

In this model structure, the residues of *Pv*PGIP2 that is known to be functionally important, have shown considerable change in SASA (Table 1), indicating that they play a key role in interaction. Four residues [V(152), S(178), Q(224) and H(271)] out of five that were identified by Leckie *et al. *[16] as interacting residues of *Pv*PGIP2 are expected to reside in the interacting surface of *Pv*PGIP2 in the *Pv*PGIP2-*Fm*PG complex, inferred on the basis of the considerable change in SASA (Table 1).

#### Docking studies of *Fm*PG-*Pv*PGIP1

Out of the ten potential candidates for models of *Pv*PGIP1-*Fm*PG complex obtained from GRAMM-X, only in one model *Pv*PGIP1 docked to the same face of *Fm*PG as was found in the model for the complex of *Pv*PGIP2-*Fm*PG (Figure 4B). In the rest of the nine models, the PGIP molecule docked to a different face of *Fm*PG indicating a gross inability of these models of *Pv*PGIP1 to block either the substrate binding site or the active site of *Fm*PG. In the selected model, whereas the portion marked with the yellow ellipse is evidently seen to be blocked in the other two complexes, the same is not the case with *Pv*PGIP1. This is in complete agreement with the experimental observation of *Pv*PGIP1 being incapable of inhibiting the PG from *Fusarium moniliforme*. This model in which *Fm*PG docks to *Pv*PGIP1 at the face similar to the one in *Pv*PGIP2 (Figure 4B) of the *Pv*PGIP2-*Fm*PG complex, is considered for further studies to confirm the inability of the protein in inhibition towards the enzyme.

#### Docking studies of *Fm*PG-*Gm*PGIP3

It has been reported that *Gm*PGIP3 possesses an inhibiting activity towards the PG of *Fusarium moniliforme *like *Pv*PGIP2. No mutational studies being available for *Gm*PGIP3, the docked structure of the *Gm*PGIP3-*Fm*PG complex was therefore selected from the top scoring ten models reported by GRAMM-X using the knowledge obtained from experimental studies carried out for *Pv*PGIP2. In the selected model of the complex (Figure 4C), *Gm*PGIP3 has blocked the active site cleft of *Fm*PG. The docked model therefore is in agreement with the experimental observation of the ability of *Gm*PGIP3 in inhibiting the enzymatic activity of *Fm*PG. The important residues V(152), S(178), Q(224) and H(271) of *Gm*PGIP3 that are also present in *Pv*PGIP2 show considerable decrease in solvent accessible surface area (Table 1) indicating their active involvement in protein-protein interaction, an identical observation that holds true for *Pv*PGIP2. Thus like *Pv*PGIP2, *Gm*PGIP3 too blocks the active site cleft and hinders the substrate binding site of *Fm*PG bearing a similar mode of competitive inhibition.

### Comparative study of protein-protein interactions in the three complexes

#### Ionic interactions

We have used PIC [28] for comparison of interactions recognized in the three complexes. Ionic interaction that is believed to play an important role in protein-protein interactions were studied for the three complexes namely *Pv*PGIP2-*Fm*PG, *Pv*PGIP1-*Fm*PG and *Gm*PGIP3-*Fm*PG. Table 2 shows that whereas this kind of interaction plays a vital role both in *Pv*PGIP2-*Fm*PG and *Gm*PGIP3-*Fm*PG complexes, it is not so for the *Pv*PGIP1-*Fm*PG complex.

**Table 2:** Ionic interaction in *Pv*PGIP2-*Fm*PG, *Pv*PGIP1-*Fm*PG *and Gm*PGIP3-Fm*PG *complexes: The ionic interaction as obtained from Protein Interaction Calculator, showing the positions of the residue pairs in *Pv*PGIP2-*Fm*PG, *Pv*PGIP1-*Fm*PG *and Gm*PGIP3-*Fm*PG docked complexes.

*Ionic interaction showing the positions of residue pairs in the Pv*PGIP2-*Fm*PG *complex for*		*Ionic interaction showing the positions of residue pairs in the Pv*PGIP1-*Fm*PG *complex for*		*Ionic interaction showing the positions of residue pairs in the Gm*PGIP3-*Fm*PG *complex for*	
***Pv*PGIP2**	***Fm*PG**	***Pv*PGIP1**	***Fm*PG**	***Gm*PGIP3**	***Fm*PG**

46	336	46	292	56	152
110	146			85	146
203	183			131	183
				225	180
				294	116

### Electrostatic surface potential of individual PGIPs and the complexes

**Figure 5:**
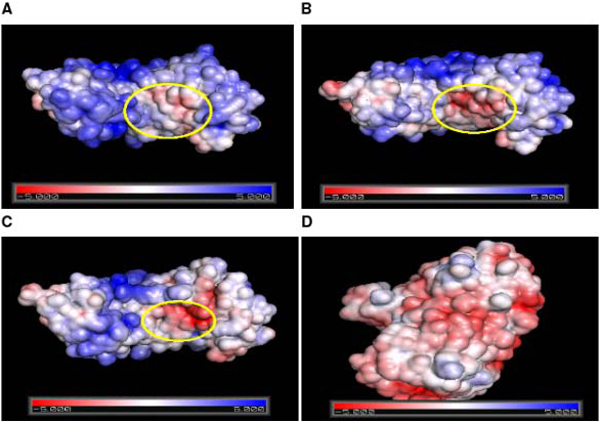
**Electrostatic surface potential of the individual proteins**. A, B, C & D show the electrostatic surface potential on solvent accessible surface around *Pv*PGIP1, *Pv*PGIP2, *Gm*PGIP3 and *Fm*PG respectively at which the surface colors are clamped at red (-5) or blue (+5). The negatively charged region marked in B and C signify the area where they interact with their partner *Fm*PG. The corresponding region in A is distinctly different with less electronegativity in surface electrostatics in comparison to B and C.

Adaptive Poisson-Boltzmann Solver (APBS) package [29] was used to generate the electrostatic surface potential for each of *Pv*PGIP1, *Pv*PGIP2, *Gm*PGIP3 and *Fm*PG, as shown in Figure 5A, B, C and 5D respectively. It is evidently clear that the electrostatic surface potential for *Pv*PGIP2 and *Gm*PGIP3 (Figure 5B &5C) show similarity along the surface at the concave face which is the site for interaction with *Fm*PG. *Pv*PGIP1 shows a distinctly different potential surface at a region in this face, even though it bears very few variations at the sequence level when compared to *Pv*PGIP2.

**Figure 6:**
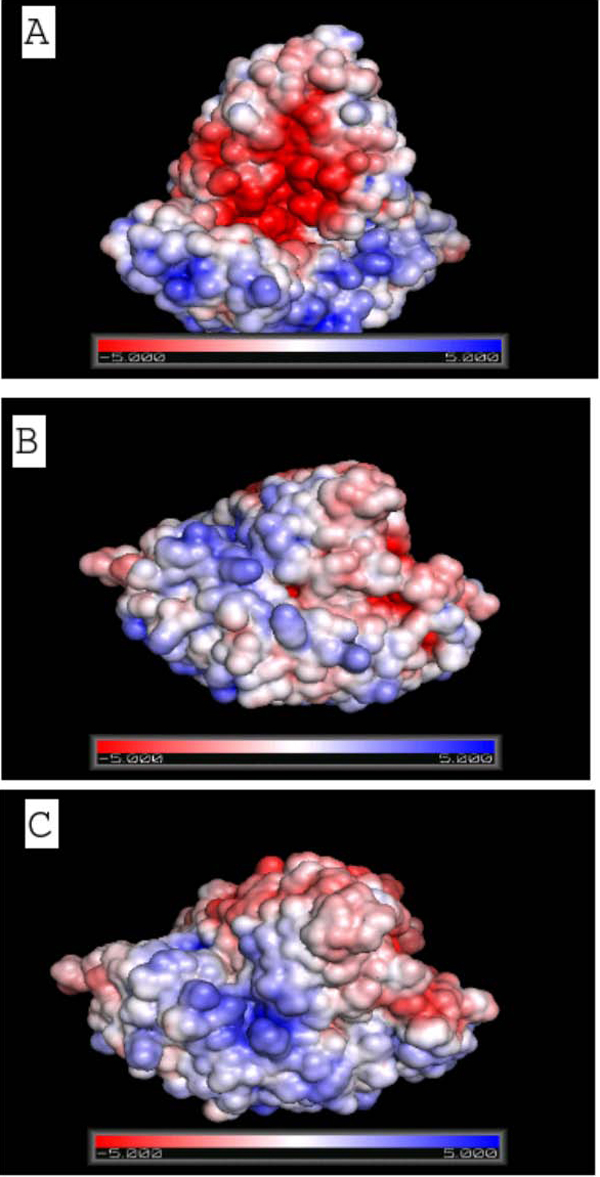
**Electrostatic surface potential of the three complexes**. A, B & C show the electrostatic surface potential on solvent accessible surface around *Pv*PGIP1-*Fm*PG, *Pv*PGIP2-*FmPG, Gm*PGIP3-*FmPG *docked complexes respectively at which the surface colors are clamped at red (-5) or blue (+5). B and C signify similar mode of binding and electrostatic interaction between the two PGIP molecules and *Fm*PG whereas the mode of interaction between *Pv*PGIP1 and *FmPG *shown in A is strikingly different from the other two.

Figure 6 shows the electrostatic potential surface and the mode of binding for the three complexes namely *Pv*PGIP1-*Fm*PG (Figure 6A), *Pv*PGIP2-*Fm*PG (Figure 6B) and *Gm*PGIP3-*Fm*PG (Figure 6C). The potential surface of the complex in *Pv*PGIP1-*Fm*PG is undoubtedly dissimilar from the other two complexes and so is the mode of binding. Interestingly, the mode of binding of *Gm*PGIP3 with *Fm*PG very much resembles that of *Pv*PGIP2 and *Fm*PG although the sequence variation between the two PGIPs is significant.

### *In silico *mutation of *Pv*PGIP2 and *Pv*PGIP2-*Fm*PG complex

*Pv*PGIP2 was mutated at six positions, with the amino acid present in *Pv*PGIP1 at that position, using WHAT IF [30]. Electrostatic surface potential was generated for all the six mutated *Pv*PGIP2 molecules. The surface drawn with the Q224K mutation showed considerable change from the wild type protein (Additional file 2). The other single mutations did not show much of a significant change in the electrostatic surface potential. It may be mentioned that when these mutated *Pv*PGIP2 molecules were docked to *Fm*PG, none of the complexes showed any resemblance to the conformation found for the *Pv*PGIP1-*Fm*PG complex, shown in Figure 6A.

When in the *Pv*PGIP2-*Fm*PG complex, Q (224) of *Pv*PGIP2 was mutated to K, using WHAT IF, the resultant complex showed quite a few of the following deviations from the complex with the wild type protein. Hard sphere contact distances [31] between heavy atoms revealed an existence of steric clash between the Nζ of Q224K of the mutated *Pv*PGIP2 and C**ε **of K(300) of *Fm*PG. Whereas the contact distance between the atoms should be more than 2.52 Å, the distance in the complex with mutation was 2.47 Å. The complex bearing the mutation also showed the absence of the predicted hydrogen bond involving Q(224) of *Pv*PGIP2 as obtained from PIC results. Even the ionic interaction that prevails in the wild type complex of *Pv*PGIP2-*Fm*PG vanishes with this single mutation. The *in silico *mutation study, confirms the importance of the Q(224) residue which has been experimentally found to give rise to a 70% reduction in the inhibition ability of the protein upon single mutation. The study also suggests probable reasons like a different electrostatic potential surface, steric clash or lack of hydrogen bonds that might explain why *Pv*PGIP1 with only eight residues has a different mode of binding to *Fm*PG.

The above *in silico *study clearly portrays how a single mutation in *Pv*PGIP2 results in disallowed hard sphere contact distances between the two protein molecules thus indicating the importance of van der Waals interaction in plant defense. Q at the 224^th ^position which is also involved in forming hydrogen bonds, plays a substantial role in this interaction. Since GRAMM-X shows that the mode of binding of *Pv*PGIP1 with *Fm*PG is different from *Pv*PGIP2 and *Gm*PGIP3 and further more since GRAMM-X has a major involvement of smoothed Lennard-Jones potential in scoring, we infer that the discrimination in recognition has a significant contribution from van der Waals interaction. Role of van der Waals forces have already been shown to be important in offering stability to several protein-ligand and in some cases DNA-ligand complexes prevailing in biological systems. Molecular Dynamics studies have shown van der Waals interaction to play a vital role in groove binding of the repressor fragment of a bacteriophage to its operator oligonucleotide [32]. In a study with proteins, it has been shown that the mouse major urinary protein (MUP) and its ligand show a binding thermodynamics that is dominated by dispersion interactions [33]. The involvement of the van der Waals forces in plant defense although not in specific recognition has also been reported. Many plants respond to pathogenic attack by producing defense proteins that bind to chitin, a polysaccharide present in the cell wall of the invading fungus or exoskeleton of infecting insects. NMR studies show the involvement of van der Waals interactions between the methyl acetamide group of the GlcNAc unit and a hydrophobic patch of the plant protein [34]. Studies on the characteristics of the sugar-binding sites in plant and animal lectin show van der Waals interaction and hydrogen bonds to contribute significantly. Selectivity for different sugars result from hydrogen bonds and unwanted recognition is prevented by steric exclusion [35]. Here in the case of plant defense through PGIP, the influence of van der Waals interaction therefore does not seem to be atypical.

In the present study, we have failed to address pertinent components like the change in the binding free energy change (ΔΔG) between the wild type and the mutated PGIPs. In order to obtain experimentally relevant information concerning the thermodynamic properties of binding, we wish to work in future, with advanced simulation methods such as free energy perturbation or thermodynamic integration to assess the binding free energy changes upon mutation of the important residues in PGIP.

## Conclusion

It has been noted from our study on sequence analysis that 50% of the sequence variation between *Pv*PGIP1 and *Pv*PGIP2 fall in the non-LRR region whereas only 15% of the variation in *Gm*PGIP3 with *Pv*PGIP2 fall in the non-LRR region. This observation suggests specific regions in PGIPs favourable for the introduction of variation in recognition of the pathogen molecules. Structural studies involving docking techniques suggest the mode of binding of the fungal enzyme *Fm*PG by PGIP2 from *Phaseolus vulgaris *to be similar to that of its homologue PGIP3 from *Glycine max*. In each case of binding, the active site of the enzyme from *Fusarium moniliforme *is being blocked by the PGIP molecule, supporting the experimentally observed phenomenon of these PGIP molecules being able to inhibit the fungal enzyme upon invasion. PGIP1 from the same plant *Phaseolus vulgaris *which is incapable of inhibiting *Fm*PG, binds to *Fm*PG in an evidently different mode, although *Pv*PGIP1 and *Pv*PGIP2 share a higher degree of sequence similarity compared to *Pv*PGIP2 and *Gm*PGIP3. Electrostatic surface potential reveals considerable difference in the interacting surface of *Pv*PGIP1 when compared to *Pv*PGIP2 or *Gm*PGIP3. Thus, electrostatic and van der Waals interactions may play a significant role in PGIPs for proper recognition and discrimination of PGs.

## Method

### Sequence analysis

The sequences of the three PGIPs, namely *Pv*PGIP1, *Pv*PGIP2 and *Gm*PGIP3 were retrieved from NCBI. We used the Pfam domain search program [23] to determine the domain architecture of these proteins. The plant specific Leucine-rich Repeats (PS_LRR) in the proteins were identified by the program PATMATDB of EMBOSS [24] using the pattern [ILVF]xx[ILVF]xx[ILVF]x [ILVF]xx[NTSC]x [ILVF] [TS]GxIPxx[ILVF]Gx as a regular expression. Pairwise alignments, one between *Pv*PGIP1 and *Pv*PGIP2 and another between *Gm*PGIP3 and *Pv*PGIP2 were carried out by the program NEEDLE of EMBOSS. All the nine repeats identified by Pfam were aligned by CLUSTALW [36] for the three proteins, individually.

### Modelling

Homology models of *Pv*PGIP1 and *Gm*PGIP3 were generated using MODELLER 9v4 [37] by satisfying the spatial restraints, based on the homology they share with the protein *Pv*PGIP2 (PDB Id: 1OGQ). The best model was selected using PROCHECK [25] based on steric correctness. Energy minimization was performed for all the structures that were involved in our studies, with the help of GROMACS 3.3.3 [38,39].

The four protein molecules namely, the models of *Pv*PGIP1 and *Gm*PGIP3 and the crystal structure of *Pv*PGIP2 and the fungal enzyme *Fm*PG (PDB Id: 1HG8) were embedded in a box of simple point charge water molecules with 1 nm separation between the box boundary and the solute. Energy minimization of solvent with the solute fixed was carried out with 500 steps of steepest descent followed by 2000 steps of conjugate gradient algorithm. After that the constraint on the solute was released and energy minimization of the whole system was carried out again with 500 steps of steepest descent followed by 2000 steps of conjugate gradient algorithm. The resulting structures were subjected to 1 ns of molecular dynamics (MD) simulations in GROMACS at 300 K. For all the simulations GROMOS96 43a1 force field was used.

The model structures of *Pv*PGIP1 and *Gm*PGIP3 have been deposited to PMDB Protein Model Database http://mi.caspur.it/PMDB/ with the PMDB identifier of PM0075783 and PM0075784 respectively.

### Docking studies

We have used GRAMM-X Protein Docking Web Server v.1.2.0 [26] for docking the three PGIP molecules to PG taken one at a time. The output PDB file that GRAMM-X produces contains 10 models ranked as the most probable prediction candidates according to the scoring function used by the program. The PGIPs acted as receptors while the PG acted as ligand in this study of protein-protein interaction. To select a model out of the ten top scoring docked complexes that GRAMM-X reported, we studied the associated change in Solvent Accessible Surface Area (SASA) of the amino acid residues in the individual molecules and when they are in the complex. We used the g_sas program in GROMACS to estimate the change in SASA for the individual residues of the proteins.

### Mutation prediction studies

We have used mutation prediction tool [40] of the WHAT IF [30] web interface to mutate Q (224) of *Pv*PGIP2 to K in the *Pv*PGIP2-*Fm*PG complex. The same mutation (Q224K) and five others that differ in *Pv*PGIP1 were carried out on *Pv*PGIP2 by using the same server.

### Determination of ionic interaction and hydrogen bonding patterns in the complexes

The Protein Interaction Calculator (PIC) [28] was used to detect the ionic interaction between the proteins in the three complexes.

### Electrostatic surface potential

Adaptive Poisson-Boltzmann Solver (APBS) is a software package for the numerical solution of the Poisson-Boltzmann equation (PBE), one of the most popular continuum models for describing electrostatic interactions between molecular solutes in salty, aqueous media. The colour coded electrostatic surface potential was drawn using the APBS (Adaptive Poisson-Boltzmann Solver) package [29] within PYMOL 0.99rc6 [41] for the three PGIP molecules, the PG molecule and the three complexes. Electrostatic surface potential was also generated for a model where a single mutation was carried out on *Pv*PGIP2 (Q224K) by WHAT IF [30,40] using the APBS package [29].

## List of abbreviations used

LRR: Leucine-rich repeat; PGIP: PolyGalacturonase Inhibiting Protein; pgip: PolyGalacturonase Inhibiting Protein coding gene; PG: PolyGalacturonase; *Pv*PGIP: *Phaseolus vulgaris *PolyGalacturonase Inhibiting Protein; *Gm*PGIP: *Glycine max *PolyGalacturonase Inhibiting Protein; *Fm*PG: *Fusarium moniliforme *PolyGalacturonase; PAMP: pathogen associated molecular pattern; HGA: homogalacturonan; PS_LRR: plant specific Leucine-rich Repeats; SASA: Solvent Accessible Surface Area; APBS: Adaptive Poisson-Boltzmann Solver; PBE: Poisson-Boltzmann equation; PIC: Protein Interaction Calculator.

## Competing interests

The authors declare that they have no competing interests.

## Authors' contributions

Both AM and SB designed the plan of work and performed the analyses. HG participated in the development of the work. SB conceived of the study and coordinated the work. All authors read and approved the final manuscript.

## Note

Other papers from the meeting have been published as part of *BMC Bioinformatics* Volume 10 Supplement 15, 2009: Eighth International Conference on Bioinformatics (InCoB2009): Bioinformatics, available online at http://www.biomedcentral.com/1471-2105/10?issue=S15.

## Supplementary Material

Additional file 1 Details of the distribution of the sequence variations in *Pv*PGIP1 and *Gm*PGIP3 with *Pv*PGIP2 over the conserved and variable portion of the repeat and the non-LRR region. The table shows the distribution of the varying residues in *Pv*PGIP1 *and Gm*PGIP3 with *Pv*PGIP2 in the LRRs and non-LRR regions. The changes that occur in the LRR region is either L/N/C of the conserved segment (LxxLxLxxNxL) or is x. They are denoted by cL, cN, cC and cX where X is any amino acid. Amino acid in the variable region of the LRR is denoted by v. Click here for file

Additional file 2 Electrostatic surface potential of *Pv*PGIP2 with a single mutation at 224. Q(224) of *Pv*PGIP2 is mutated to K which is the residue found in *Pv*PGIP1. The electrostatic surface potential changes considerably with a single mutation when compared to Figure 5B. Experimental studies on this single mutation have shown a 70% reduction in the inhibition ability of *Pv*PGIP2. Click here for file
