# Supplementary material for: Comparative study of protein-protein interaction observed in PolyGalacturonase-Inhibiting Proteins from Phaseolus vulgaris and Glycine max and PolyGalacturonase from Fusarium moniliforme
Source: BMC Genomics. 2009 Dec 3;10(Suppl 3):S19. doi: 10.1186/1471-2164-10-S3-S19 (PMC2788371; doi:10.1186/1471-2164-10-S3-S19)
Supplement: Additional file 1 — Details of the distribution of the sequence variations in PvPGIP1 and GmPGIP3 with PvPGIP2 over the conserved and variable portion of the repeat and the non-LRR region. The table shows the distribution of the varying residues in PvPGIP1 and GmPGIP3 with PvPGIP2 in the LRRs and non-LRR regions. The changes that occur in the LRR region is either L/N/C of the conserved segment (LxxLxLxxNxL) or is x. They are denoted by cL, cN, cC and cX where X is any amino acid. Amino acid in the variable region of the LRR is denoted by v. [file 1471-2164-10-S3-S19-S1.pdf]

Additional file 1: Details of the distribution of the sequence variations in *Pv*PGIP1 and *Gm*PGIP3 with *Pv*PGIP2 over the conserved and variable portion of the repeat and the nonLRR region

The table shows the distribution of the varying residues in *Pv*PGIP1 *and Gm*PGIP3 with *Pv*PGIP2 in the LRRs and nonLRR regions. The changes that occur in the LRR region is either L/N/C of the conserved segment (LxxLxLxxNxL) or is x. They are denoted by cL, cN, cC and cX where X is any amino acid. Amino acid in the variable region of the LRR is denoted by v.

Residue No	***Residue type and their position with respect to the LRR pattern (PvPGIP2)***	***Residue type and their position with respect to the LRR pattern (PvPGIP1)***	***Residue type and their position with respect to the LRR pattern (GmPGIP3)***
54	N (**c**X)	unchanged	D (**c**X)
59	G (**c**X)	unchanged	D (**c**X)
60	L (**c**N)	H (**c**N)	unchanged
67	P (**v**)	unchanged	S (**v**)
70	S (**v**)	unchanged	P (**v**)
72	L(**v**)	unchanged	I (**v**)
82	Y (**c**X)	unchanged	S (**c**X)
84	G (**c**X)	unchanged	S (**c**X)
85	G (insertion)	unchanged	R (**c**X)
86	I (**c**X)	unchanged	T (**c**N)
87	N (**c**N)	unchanged	P (insertion)
88	N (**c**X)	unchanged	T (**c**X)
90	V (**v**)	unchanged	I (**v**)
92	P (**v**)	unchanged	Q (**v**)
95	P (**v**)	unchanged	S (**v**)
104	H (**c**X)	unchanged	R (**c**X)
116	A (**v**)	unchanged	P (**v**)
136	A (**c**X)	unchanged	T (**c**X)

Residue No	*Residue type and their position with respect to the LRR pattern (Pv***PGIP2***)*	*Residue type and their position with respect to the LRR pattern (Pv***PGIP1***)*	*Residue type and their position with respect to the LRR pattern (Gm***PGIP3***)*
140	T (**v**)	unchanged	K (**v**)
143	P (**v**)	unchanged	A (**v**)
145	I (**v**)	unchanged	L (**v**)
152	V (**c**X)	G (**c**X)	Unchanged
160	R (**c**X)	unchanged	Q (**c**X)
178	S (**c**X)	A (**c**X)	Unchanged
192	P (**v**)	unchanged	A (**v**)
219	S (**v**)	unchanged	T (**v**)
220	D (non-LRR)	unchanged	E (non-LRR)
224	Q (non-LRR)	K (non-LRR)	Unchanged
227	H (non-LRR)	unchanged	L (non-LRR)
232	S (non-LRR)	unchanged	M (non-LRR)
266	Q (**v**)	unchanged	A (**v**)
271	H (**c**X)	Q (**c**X)	unchanged
273	L (**c**L)	unchanged	F (**c**L)
279	N (**c**X)	unchanged	D (**c**X)
290	L (**v**)	unchanged	M (**v**)
291	Q (non-LRR)	K (non-LRR)	Unchanged
297	A (non-LRR)	S (non-LRR)	S (non-LRR)
311	A (non-LRR)	S (non-LRR)	P (non-LRR)
