# Supplementary material for: Comparative study of protein-protein interaction observed in PolyGalacturonase-Inhibiting Proteins from Phaseolus vulgaris and Glycine max and PolyGalacturonase from Fusarium moniliforme
Source: BMC Genomics. 2009 Dec 3;10(Suppl 3):S19. doi: 10.1186/1471-2164-10-S3-S19 (PMC2788371; doi:10.1186/1471-2164-10-S3-S19)

Additional file 2: Electrostatic surface potential of PvPGIP2 with a single mutation at 224 Q(224) of PvPGIP2 is mutated to K which is the residue found in PvPGIP1.

The electrostatic surface potential changes considerably with a single mutation when compared to Figure 5B. Experimental studies on this single mutation have shown a 70% reduction in the inhibition ability of PvPGIP2.

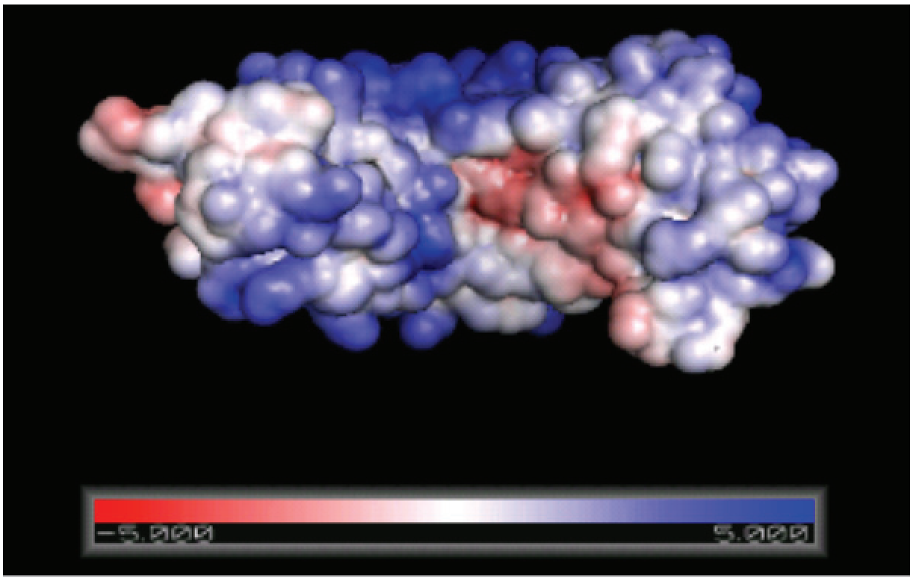

Supplement: Additional file 2 — Electrostatic surface potential of PvPGIP2 with a single mutation at 224. Q(224) of PvPGIP2 is mutated to K which is the residue found in PvPGIP1. The electrostatic surface potential changes considerably with a single mutation when compared to Figure 5B. Experimental studies on this single mutation have shown a 70% reduction in the inhibition ability of PvPGIP2. [file 1471-2164-10-S3-S19-S2.pdf]
